# In Situ CO_2_‐Induced Hydrogels for Enhancing Carbon Sequestration in Saline Aquifers

**DOI:** 10.1002/advs.77958

**Published:** 2026-09-27

**Authors:** Fuchuan Liu, Xinjie Luo, Dianguo Wu, Xuezhi Zhao, Yan Zhang, Yujun Feng

**Affiliations:** ^1^ Polymer Research Institute, State Key Laboratory of Advanced Polymer Materials Sichuan University Chengdu People's Republic of China

**Keywords:** CO_2_ sequestration, CO_2_‐triggered hydrogels, deep eutectic solvents, saline aquifers

## Abstract

Carbon sequestration in saline aquifers underpins global net‑zero emission goals, yet existing CO_2_ storage capacity remains inadequate, and conventional approaches exhibit limited adaptability to reservoir environment. Here, we introduce smart, injectable soft materials based on CO_2_‑triggered hydrogels integrated with tailored deep eutectic solvents (DESs) to boost sequestration. The hydrogel forms upon CO_2_‑triggered protonation of erucamine (a C_22_‑tailed tertiary amine) within its aqueous dispersion, which promotes electrostatic interactions and hydrophobic self‑assembly. The DESs (choline chloride/polyethylene glycol‑200/boric acid) further enhance system functionality. In situ gelation and flow behavior of these soft materials is validated via visualization and rheology under simulated reservoir conditions (45°C, 10 MPa, 4500 mg·L^−1^ NaCl). Sequestration assays reveal that a 3.0 wt% erucamine‐based hydrogel increases CO_2_ storage by 31.8% relative to brine; incorporation of 5.0 wt% DESs to form a hybrid hydrogel further elevates storage to 52.9%. Notably, the hydrogel retains structural integrity with no detectable CO_2_ leakage throughout a 146‑day monitoring period at 45 °C. The improved CO_2_ sequestration stems from three synergistic mechanisms: erucamine‐CO_2_ reaction, hydrogel physical entrapment, and DESs‐promoted solubility. This work establishes a quantifiable and scalable soft material platform that offers a transformative route for advancing CO_2_ sequestration in saline aquifers.

## Graphical abstract

A novel integrated method that combines in situ CO_2_‐induced phase‐transition hydrogels formed by a C_22_‐tailed tertiary amine dispersion with tailored deep eutectic solvents to increase CO_2_ sequestration in saline aquifers.

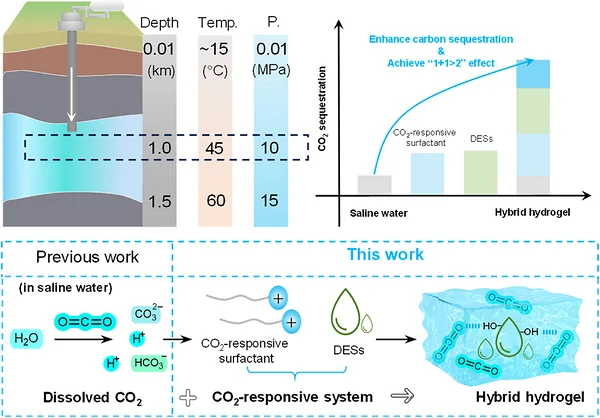

## 1 Introduction

Carbon capture, utilization, and sequestration (CCUS) technologies stand as a pivotal pathway to attain global CO_2_ emissions targets and advance sustainable development goals [1, 2, 3, 4]. The Net Zero Emissions Scenario mandates annual CO_2_ sequestration capacity of ≈1 billion tons by 2030 to align with the Paris Agreement's 1.5°C warming limit [5]. Saline aquifers emerge as the most scalable storage option [6, 7], boasting widespread distribution and immense theoretical potential (≈1,914 Gt CO_2_) [8], yet actual global sequestration remains 0.1% of this capacity [9, 10, 11]. Heterogenous formations [12, 13], complex CO_2_ migration [14], and pervasive leakage risks [15, 16] plague conventional approaches, creating an urgent need for innovative materials and strategies to boost storage efficiency and reliability in saline aquifers.

Global CO_2_ sequestration in saline aquifers currently is estimated to be less than totals 50.5 Mt annually, varying with formation structure, environmental conditions, and salinity range [16, 17]. Under high‐temperature, high‐pressure (HTHP) reservoir conditions [18, 19, 20, 21], brine's maximum CO_2_ storage capacity rarely exceeds 60 g·L^−1^: numerical estimates cite ≈30 g·L^−1^ CO_2_ at 40 °C and 12 MPa [22], while experiments report ≈35 g·L^−1^ at 59 °C and 10.38 MPa [23]. Large‐scale projects such as Sleipner, Quest, and Tomakomai each store less than 1 Mt per year [24], underscoring that conventional methods alone cannot bridge the capacity gap.

To address this, physical, chemical, and biological strategies have been explored [25, 26, 27]. Physical approaches (e.g., water‐alternating‐gas injection [28], well placement optimization [29], and injection pressure management [30]) improve sweep efficiency but falter in heterogeneous formations [13]. Chemical methods utilize surfactants, polymers, or nanoparticles to reduce CO_2_ mobility or enhance solubility trapping [26, 31, 32]. Surfactant‐based systems show promise—foam injection increased sequestration rate from 11.40% to 52.35% [33], in situ foam boosted storage resources by 17% [34], and surfactant‐nanomaterial composites raised sequestration from 9.2% to 26.4% [35]. However, practical field application of conventional chemical agents is significantly constrained by the harsh native conditions of deep saline aquifers, such as high salinity, elevated reservoir temperatures, and mineral‑rock interfaces [7, 9]. For example, foam systems readily degrade under high‑temperature‑high‑pressure (HTHP) environments [32, 36]; high salinity can trigger chemical‑agent precipitation, and mineral‑rock surfaces may adsorb active components and diminish their performance [37, 38]. To mitigate these drawbacks, prior efforts have pursued multiple mitigation strategies, including rational molecular‑structure tailoring of chemical agents, advanced injection schemes, and blended chemical formulations [35, 37]. Moreover, biological methods suffer from slow reaction kinetics, microbial survival constraints, and pore clogging risks [39, 40].

CO_2_‐responsive materials, including thickening polymers [41, 42], foams [43], and hydrogels [44, 45], have gained traction for geological storage due to reversible physicochemical property shifts upon CO_2_ exposure [46, 47, 48, 49]. CO_2_‐responsive hydrogels show potential for mobility control and leakage mitigation [50, 51], yet their application in saline aquifer sequestration remains nascent, demanding further development for realistic reservoir conditions.

Deep eutectic solvents (DESs), green solvents with simple synthesis, low‐cost feedstocks, and non‐toxic/non‐volatile characteristics [52], represent another emerging class for CO_2_ management [53], predominantly focusing on CO_2_ capture from flue gases [54, 55]. Composed of hydrogen‐bond donors (HBDs) and acceptors (HBAs), DESs exhibit CO_2_ uptake across diverse pressures and temperatures [56], suggesting HTHP compatibility. However, their use in saline aquifer sequestration remains largely unexplored.

Accurate in situ sequestration capacity quantification is also critical. Conventional ex situ techniques, including gravimetric analysis [57], gas chromatography [58], and titration [59], introduce environmental perturbations and uncertainties (Table S1), highlighting the need for robust real‐time measurement platforms under authentic geological conditions.

To tackle these challenges, this study presents a soft‑material platform combining CO_2_‐induced hydrogels and DESs to enhance sequestration performance and enable quantitative evaluation. Hydrogels formed via CO_2_‐triggered self‐assembly of *N*‐erucamidopropyl‐*N,N*‐dimethylamine (erucamine) into viscoelastic wormlike micelles (WLMs) [60], while DESs were synthesized from choline chloride, boric acid, and PEG200. Under simulated reservoir conditions (45°C, 10 MPa CO_2_), in situ visualization and rheological test confirmed shear‐reversible gelation, which is conducive to migration in the formation. A customized device quantified sequestration capacity: erucamine concentration increases from 1.0 to 5.0 wt% elevates capacity from 110.5 to 170.2 g·L^−1^, and 5.0 wt% DESs addition to 3.0 wt% erucamine yields a 52.9% synergistic enhancement. The precursor solution is verified to possess favorable injectability and can form hydrogels in situ. The hydrogel maintains structural stability over a 146‑day monitoring period with no detectable CO_2_ leakage, demonstrating reliable sequestration stability. Spectroscopic and microscopic analyses reveal strengthened hydrogen‑bonding interactions and a denser micellar network within the system. This study develops a novel soft‐material platform with quantifiable performance and scalable potential for saline‐aquifer carbon sequestration, offering new insights into the rational design of responsive gel systems.

## 2 Results and Discussion

### 2.1 Design Strategy for Augmented CO_2_ Storage in Saline Aquifers

**FIGURE 1:**
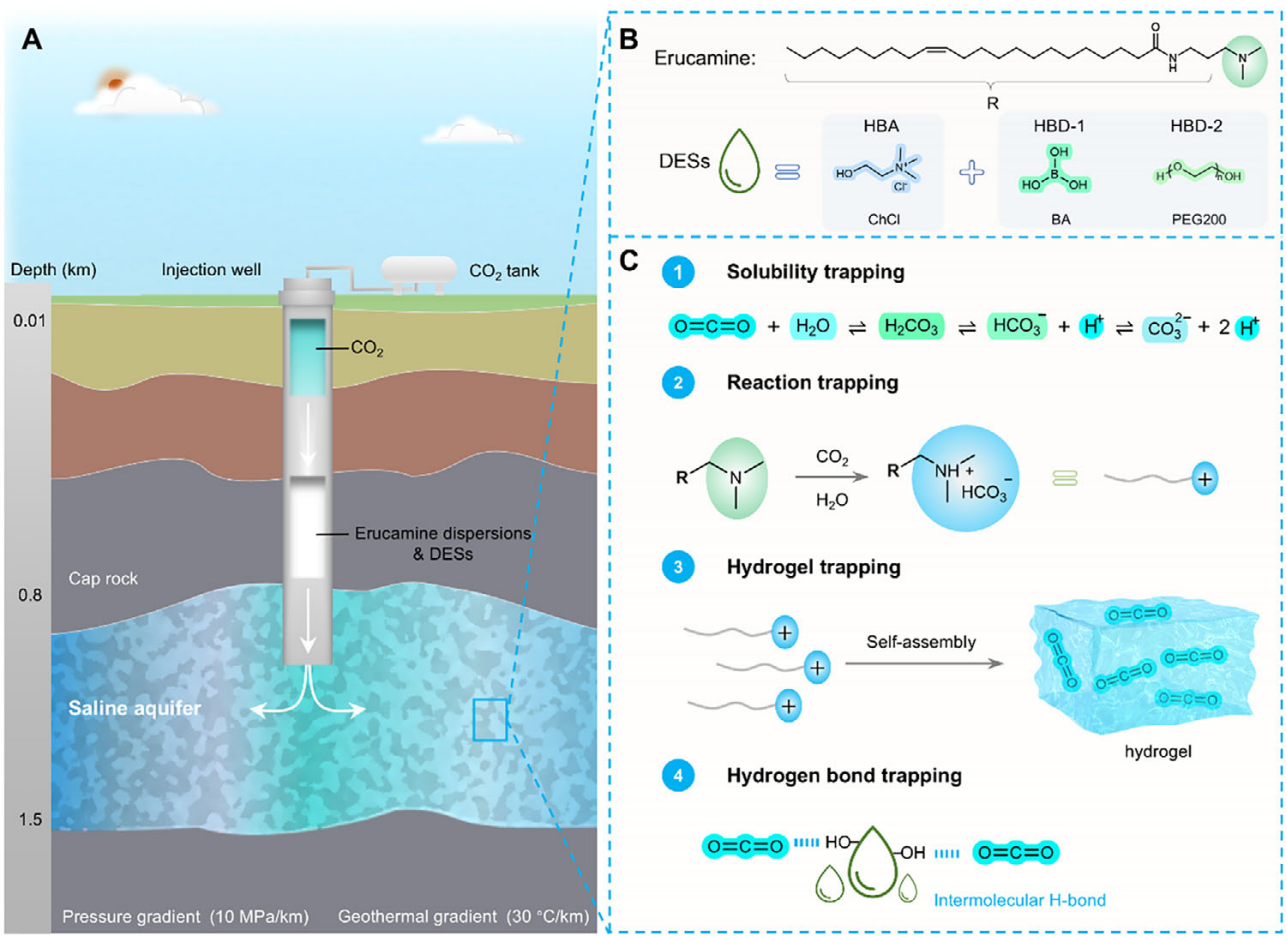
Enhanced carbon sequestration in saline aquifer via synergistic interactions between CO_2_‐induced hydrogels and DESs. (A) Schematic of the CO_2_ sequestration scenario. (B) Molecular structures of erucamine and DESs components. (C) Fourfold interaction mechanisms facilitating enhanced CO_2_ retention.

To enhance CO_2_ sequestration in deep saline formations, this work integrates tailored materials with a staged injection protocol. As illustrated in Figure 1, the proposed approach departs from conventional direct CO_2_ injection: a hybrid system containing erucamine and DESs is first delivered to the target zone—identified as the optimal interval for CO_2_ storage at depths of 800–1500 m [61] —followed by controlled alternating CO_2_ injection (Figure 1A).

Based on the molecular architecture of erucamine and DESs (Figure 1B) and native aquifer conditions, a mechanistic hypothesis is proposed: upon CO_2_ introduction, four distinct interaction pathways —solubility trapping, reaction trapping, hydrogel trapping, and hydrogen‐bond trapping—operate synergistically within the formation (Figure 1C) to reinforce CO_2_ immobilization. Subsequent sections investigate and validate these mechanisms, elucidating the fundamental processes underpinning secure carbon storage.

### 2.2 In Situ CO_2_‐Induced Gelation of Erucamine Dispersions

The efficacy of the proposed strategy for enhanced carbon storage was first evaluated by examining in situ hydrogel formation under simulated saline aquifer conditions. Gelation was monitored via two complementary visualization techniques at representative reservoir parameters (5.0 wt% erucamine, 1,000 m depth, 45°C, 10 MPa, 4,500 mg·L^−1^ NaCl).

**FIGURE 2:**
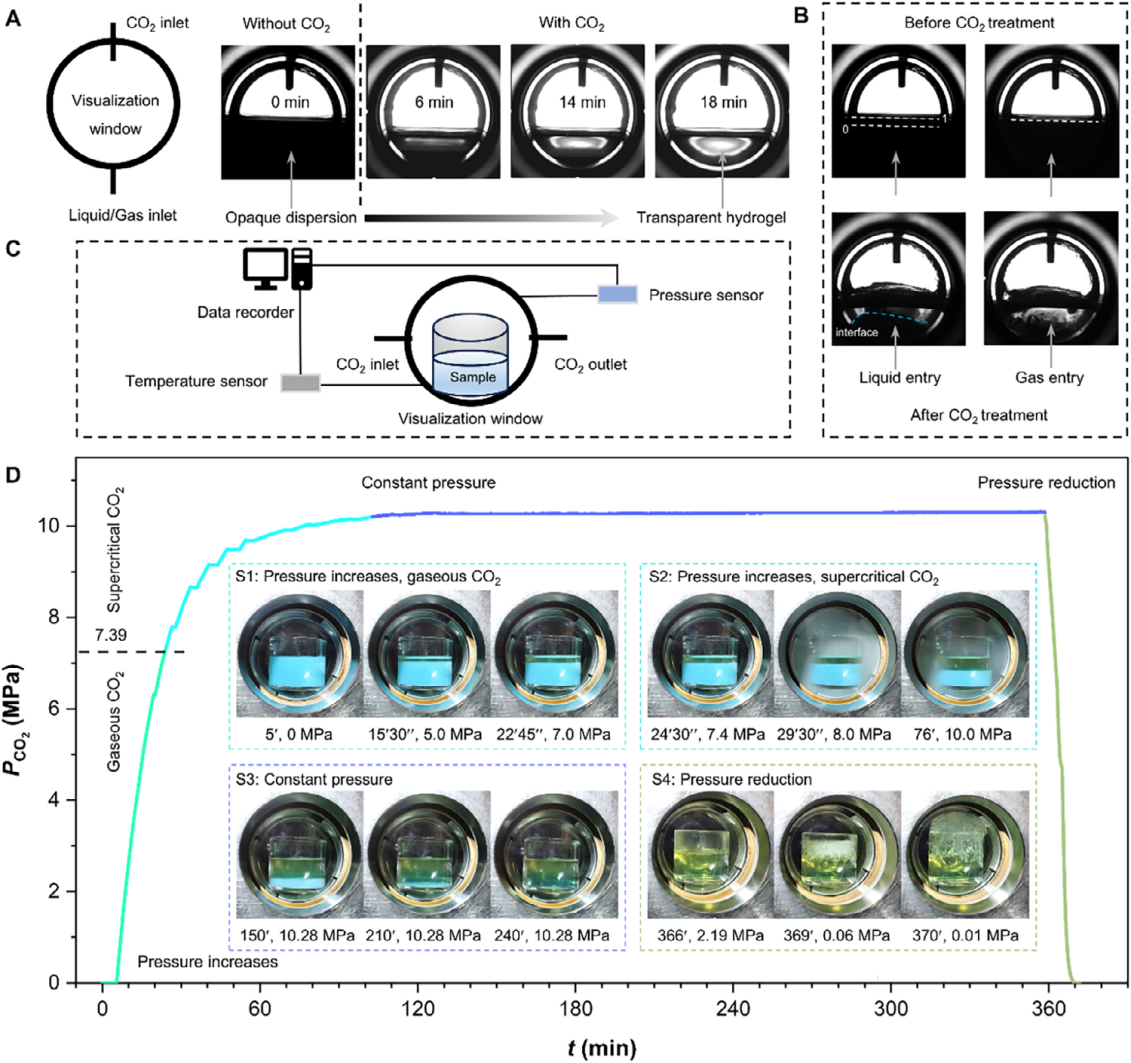
In situ visualization of CO_2_‐induced hydrogel formation at 45°C and 10 MPa CO_2_. (A) Schematic cross‐section of the visualization device (left) and corresponding fluid states (right) under ambient (no CO_2_) and high‐pressure CO_2_ conditions (images from real‐time recordings). (B) Phase contrast between the initial erucamine dispersion (liquid) and the CO_2_‐treated system (gel) following fluid reinjection. White dashed line (0) marks the initial liquid level, line (1) indicates the level after injection; blue dashed line highlights the phase interface. (C) Schematic of the custom high‐temperature, high‐pressure visualization setup. (D) Time‐dependent morphological evolution of a 5.0 wt% erucamine dispersion (containing 4,500 mg·L^−1^ NaCl) at 45°C and 10 MPa CO_2_.

Initial phase behavior was observed using a commercial HTHP visualization system (Figure S1A). Figure 2A displays the visualization cell fitted with gas‑liquid inlet and outlet ports. Under ambient conditions (prior to CO_2_ exposure), the erucamine dispersion is an opaque dark liquid. As CO_2_ pressure rises to 10 MPa, this opaque mixture gradually turns colorless and transparent over time (Figure 2A, Movie S1). To verify that this transparent state represents hydrogel formation, reinjection tests were carried out. Identification relies on contrasting interfacial behavior among liquid dispersion, soft‑solid hydrogel, and coexisting fluid phases. No discrete phase boundaries form for dispersion‑liquid or dispersion‑gas combinations; by comparison, well‑defined interfaces develop along hydrogel‑liquid and hydrogel‑gas boundaries. As illustrated in Figure 2B, reinjecting liquid and gas into the original erucamine dispersion yields no observable phase boundary (white dashed lines, Figure 2B, top). For the CO_2_‑processed transparent sample, however, distinct interfaces (blue dashed line, Figure 2B, bottom) and entrapped bubbles emerge upon fluid reinjection. Such incompatibility signatures confirm conversion of the starting dispersion into a hydrogel. These observations directly demonstrate CO_2_‐triggered hydrogelation and establish a straightforward physical method for phase identification without ancillary analytical tools.

While informative, the commercial setup lacked real‐time monitoring capabilities, relying on post‐process reinjection. To enable continuous in situ analysis, a custom HTHP reaction‐monitoring system (Figure 2C, Figure S1B) was developed, capturing the temporal morphological evolution of a 5.0 wt% erucamine dispersion under constant 10 MPa CO_2_ (Figure 2D, Movie S2).

The gelation process unfolded in four distinct stages: (i) Gaseous CO_2_ pressurization (green line, Figure 2D): Gelation initiated at the gas‐liquid interface, propagating downward with gradual CO_2_ absorption. (ii) Transition to supercritical CO_2_ (cyan line, Figure 2D): Above the critical pressure (7.39 MPa), CO_2_ entered a supercritical state, accelerating the reaction. Under static conditions, however, the advancing gel front impeded CO_2_ diffusion, slowing progression (e.g., the transition from 1/4 to 1/2 gelation required ∼ 46.5 min). (iii) Constant pressure maintenance (blue line, Figure 2D): After over four hours at 10 MPa, the reaction neared completion, yielding a homogeneous yellow‐green gel. (iv) Depressurization (yellow‐green line, Figure 2D): Pressure release induced viscous surface bubbles, confirming hydrogel formation. While some CO_2_ escaped during this stage (Movie S3)—causing localized collapse—the persistent coloration indicated substantial residual CO_2_ retention within the gel matrix.

These macroscopic transformations confirm effective CO_2_ sequestration within the hydrogel under HTHP conditions, aligning with the solubility, reaction, and hydrogel trapping mechanisms outlined in Figure 1C. Notably, CO_2_ release during depressurization underscores a limitation of conventional sampling: abrupt pressure changes can perturb the gel's native state and liberate sequestered CO_2_, potentially underestimating trapping capacity. Collectively, these results demonstrate that stable hydrogels form in situ upon CO_2_ exposure under simulated reservoir conditions.

### 2.3 Flow Behavior and Dynamic Gelation of Erucamine Dispersions

**FIGURE 3:**
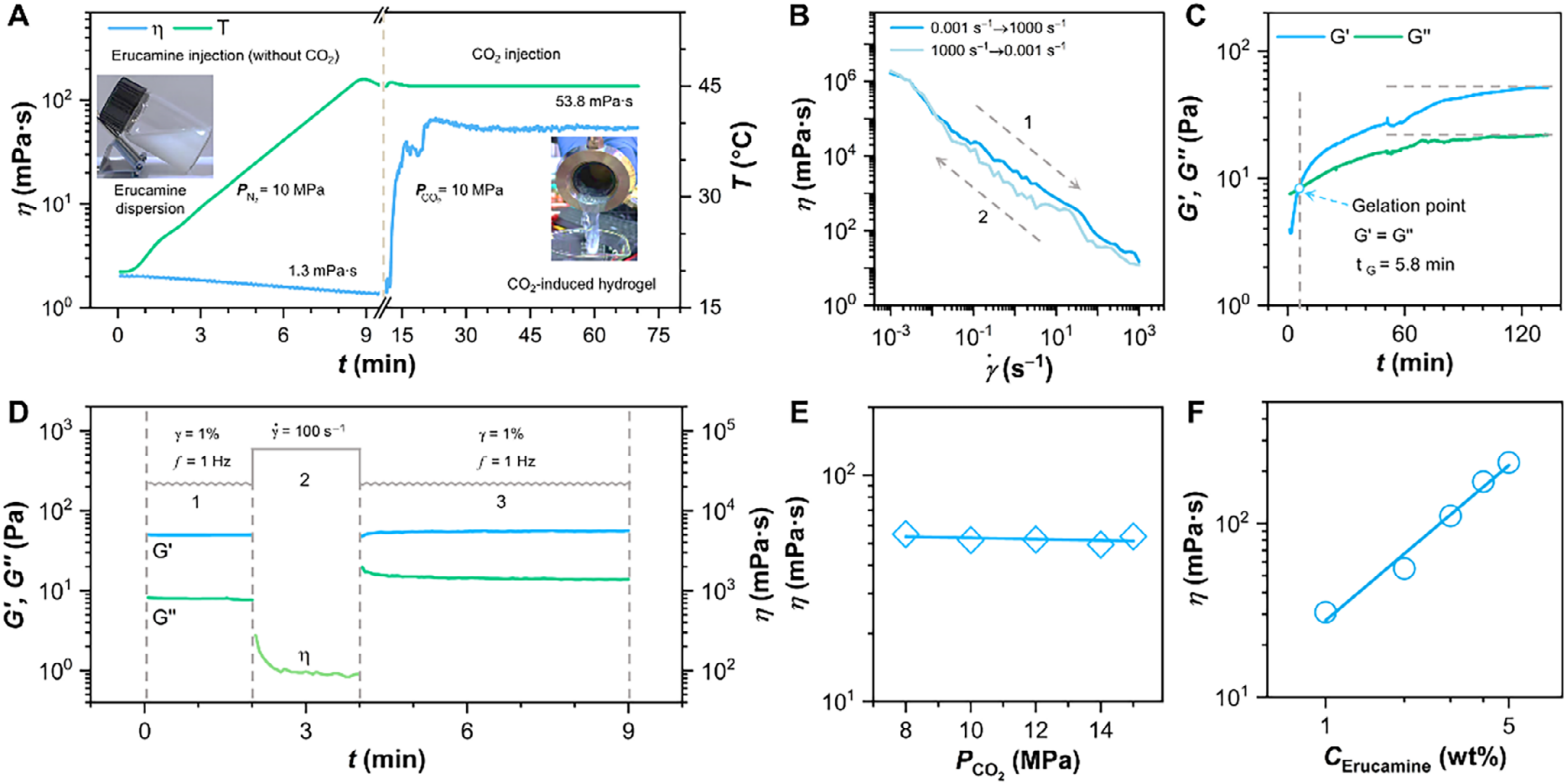
Rheological characterization of erucamine dispersions under simulated saline aquifer conditions. (A) Apparent viscosity (*η*, blue) and temperature (*T*, green) profiles of a 2.0 wt% dispersion at 100 s^−1^ under 45°C, 10 MPa, illustrating injectivity and CO_2_‐triggered gelation. Insets show the erucamine dispersion (left) and transparent gel formed after CO_2_ exposure (right). (B) Shear‐thinning behavior of the CO_2_‐induced hydrogel (45°C, 10 MPa CO_2_) demonstrated via cyclic shear loops. (C) Temporal evolution of storage (*G*′, blue) and loss (*G*″, green) moduli for a 2.0 wt% dispersion during an oscillatory time sweep under 45°C, 10 MPa CO_2_. (D) Structural recovery assessed through oscillatory–shear–oscillatory sequence. Stages 1 and 3 (oscillation): moduli under quiescent conditions; stage 2 (rotation): viscosity under high shear. The grey line denotes the test protocol, with experimental parameters noted above. Steady‐shear viscosity of CO_2_‐induced hydrogels as a function of (E) pressure and (F) erucamine concentration at 45°C and 100 s^−1^, highlighting shear‐resistant behavior.

Previous experiments characterized erucamine dispersion gelation under static saline aquifer‐mimicking conditions. Given that effective carbon sequestration inherently involves fluid transport, the rheological response during flow was also investigated. Using an HTHP rheometer configured to emulate subsurface porous media (Figure S2), flow characteristics and gelation kinetics of erucamine dispersions were examined. The applied thermal profile comprised two stages (Figure 3A): a heating ramp under N_2_ atmosphere (mimicking the geothermal gradient during injection from surface to reservoir depth) followed by an isothermal plateau under CO_2_‐saturated conditions.

*Injectivity and In Situ Gelation*. Injectability was evaluated by monitoring the apparent viscosity (*η*) of a 2.0 wt% dispersion. As the temperature increased from 20°C to 45°C, *η* declined from 2.0 to 1.3 mPa·s (Figure 3A), confirming favorable flow characteristics prior to CO_2_ exposure. Upon CO_2_ introduction, *η* surged to 53.8 mPa·s at 100 s^−1^, accompanied by a distinct sol–gel transition (Figure 3A, insets). This trend held across concentrations (Figure S3): a 1.0 wt% dispersion yielded only a viscous fluid (*η* = 22.8 mPa·s), while a 5.0 wt% system attained a final viscosity of 205.2 mPa·s. Intriguingly, even at the highest concentration, initial viscosity remained below 10 mPa·s, preserving injectability. These findings demonstrate that erucamine dispersions satisfy a critical operational requirement—low viscosity during injection followed by substantial thickening upon CO_2_ contact—a prerequisite for effective fluid diversion and CO_2_ entrapment.

*Shear‐Thinning and Reversible Flow*. Pore‐scale heterogeneity in saline aquifers exposes gels to a wide range of shear rates, prompting investigation of the shear‐dependent rheology of CO_2_‐induced hydrogels (2.0 wt%; Figure 3B, Figure S4). The material exhibited pronounced shear‐thinning, a hallmark of non‐Newtonian behavior: at shear rates below 0.1 s^−1^, viscosity exceeded 10^4^ mPa·s, while the rates above 100 s^−1^ remarkably reduced viscosity— facilitating propagation through porous media. The absence of a measurable zero‐shear viscosity (*η*_0_) further confirmed CO_2_‐triggered gelation [50], in contrast to the 1.0 wt% dispersion, which displayed a clear *η*_0_ (Figure S4). Cyclic shear tests (Figure 3B) revealed complete reversibility with minimal hysteresis, indicating the gel network readily reforms after shear‐induced disruption. This allows the hydrogel to migrate deep in the saline aquifers and is beneficial to pumping. Moreover, shear‐thinning behavior enables deep reservoir penetration followed by structural recovery, a critical attribute for stabilizing sequestered CO_2_.

*Gelation Kinetics*. Oscillatory time‐sweep measurements elucidated gel formation dynamics (Figure 3C). Both storage (*G*′) and loss (*G*″) moduli increased progressively until equilibrium. Initially (t < 5.8 min), the system displayed liquid‐like behavior (*G*″ > *G*′). A distinct rheological crossover ensued, after which *G*′ exceeded *G*″, signaling the establishment of a continuous gel network [62, 63]. This transition confirms in situ CO_2_‐mediated gelation under saline aquifer‐relevant conditions. Furthermore, gelation time is governed by temperature, pressure, and erucamine loading. Within the typical temperature range of saline aquifers (20  C–60 °C), higher temperatures expedite molecular diffusion and proton‑transfer reactions and thus curtail gelation time. Nevertheless, overly elevated temperatures (>75 °C) may disintegrate the wormlike micellar network, lower system viscosity, and even suppress hydrogel development [50]. Gelation proceeds far more slowly under gaseous low‑pressure CO_2_ relative to supercritical CO_2_, consistent with our earlier findings [50, 60]. Increasing erucamine content speeds up gel build‑up, where a shorter viscosity‑ramp period in Figure S3 denotes faster gel evolution.

*Structural Recovery After Shear*. Network regeneration capability was probed via an oscillatory–shear–oscillatory sequence (Figure 3D). In stage 1 (oscillation, 1% strain, 1 Hz), *G*′ > *G*″ confirmed solid‐like behavior at rest. During high‐shear stage 2, viscosity declined sharply, reflecting temporary network rapture. Upon returning to oscillatory conditions in stage 3, moduli recovered rapidly, underscoring robust self‐reorganization. This swift recovery supports repeated shear tolerance and sustained performance under dynamic subsurface flow.

*Pressure and Concentration Dependence*. Long‐term containment requires gel stability across varying pressures and concentrations. Steady‐shear viscosity measurements (Figure 3E and F, Figure S5) showed two key trends: at fixed concentration (2.0 wt%), hydrogel viscosity remained essentially constant (53 ± 3 mPa·s) over the investigated pressure range; at constant CO_2_ pressure (10 MPa), viscosity increased monotonically with erucamine concentration. This behavior stems from enhanced intermolecular interactions—higher concentration provides more protonation sites upon CO_2_ exposure, amplifying electrostatic repulsion and hydrophobic association to elevate viscosity [64].

Collectively, these experiments demonstrate that erucamine dispersions possess three essential attributes for subsurface application: low initial viscosity facilitating injection, shear‐thinning and reversible flow enabling deep propagation, and in situ CO_2_‐triggered gelation with rapid structural recovery. These characteristics align closely with the operational demands of effective carbon sequestration in saline aquifers.

### 2.4 CO_2_ Sequestration Capacity

**FIGURE 4:**
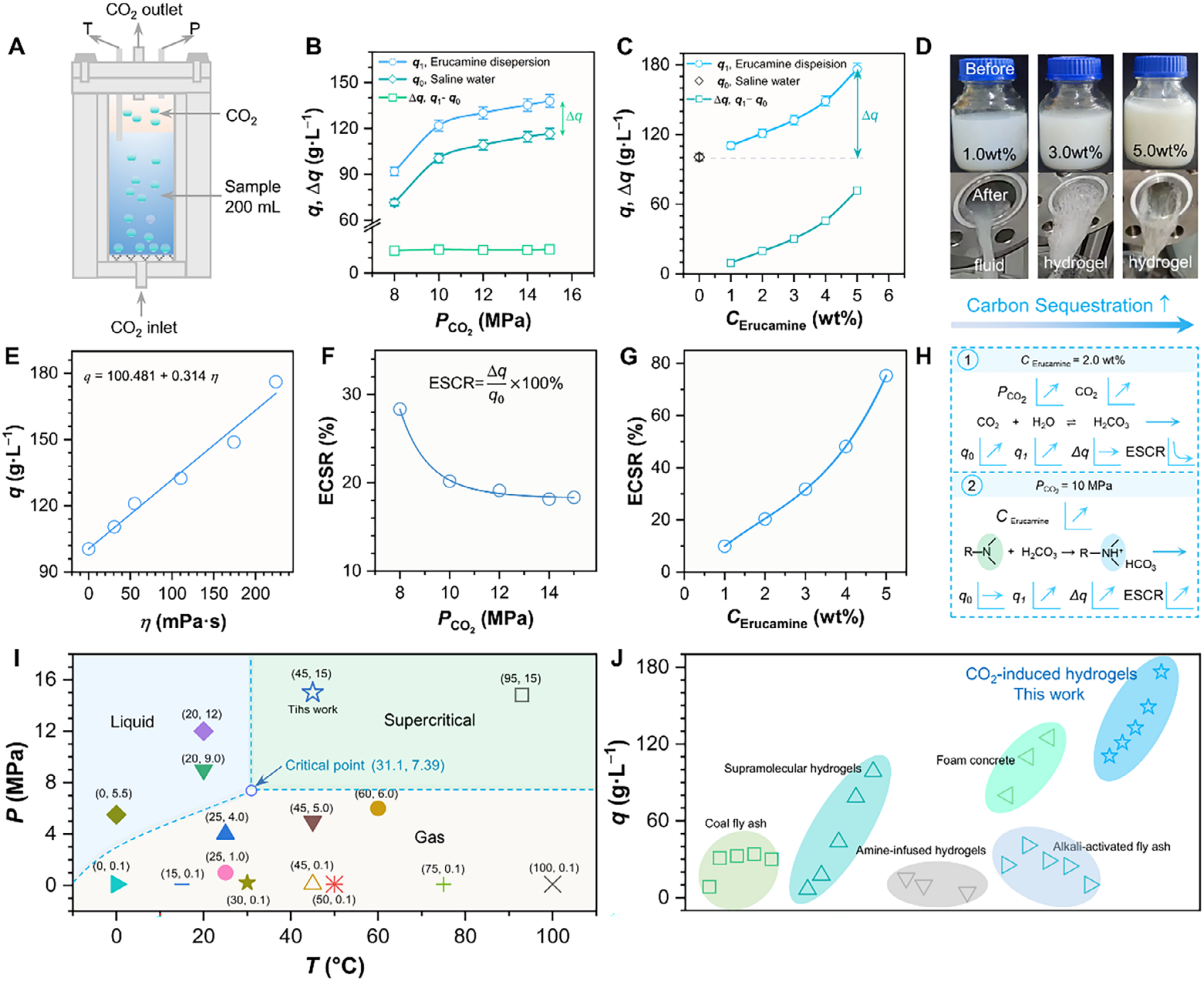
Quantitative assessment of CO_2_ sequestration performance. (A) Experimental configuration simulating aquifer conditions for CO_2_ storage studies. (B) Sequestration capacity (*q*) as a function of pressure at 45°C for 2.0 wt% erucamine dispersion (light blue circles) and a 4,500 mg·L^−1^ NaCl solution (cyan diamonds), with enhancement factor (Δ*q*) (green squares). (C) Concentration‐dependent sequestration capacity at 10 MPa and 45°C for erucamine dispersions (light blue squares) and a reference NaCl solution (black diamond), with the associated Δ*q* (cyan circles). Error bar: 3%. (D) Macroscopic phase behavior of erucamine dispersions after CO_2_ exposure, showing transition from free‐flowing liquid to viscoelastic gel. (E) Correlation between hydrogel viscosity (*η*) and *q* (light blue circles) with linear regression (blue line, R^2^ = 0.972). (F) Enhanced CO_2_ sequestration rate (ECSR) versus pressure for 2.0 wt% erucamine dispersion. (G) ECSR as a function of erucamine concentration at 10 MPa. (H) Schematic of the mechanisms underlying CO_2_ sequestration enhancement. (I) Comparison of operational conditions (temperature vs. pressure) in reported CO_2_ capture and storage studies by materials (≤ 100°C) [65, 66, 67, 68, 69, 70] with this work (blue star). (J) Comparative evaluation of CO_2_ sequestration capacity (*q*) across different materials: direct mineral carbonation of coal fly ash (conditions: 60°C, 3 MPa); bile salt hydrogel (25°C, 0.1 MPa); amine‑infused hydrogel (25°C, 1 bar); foam concrete (ambient conditions) [71, 72, 73, 74, 75]; and this work (45°C, 10 MPa).

With gelation behavior and aquifer compatibility established, the CO_2_ sequestration potential of erucamine dispersions was quantitatively evaluated. A bespoke experimental apparatus (Figure S6) was developed to precisely measure CO_2_ uptake in both liquid and gel phases, advancing prior designs through integration of a high‐precision mass flowmeter (±0.01%) for real‐time CO_2_ mass tracking and an HTHP reactor (Figure 4A) enabling acute control of temperature and pressure to replicate aquifer conditions. The system supports synchronized data acquisition (Figure S7), with pressure‐dependent CO_2_ density data (Figure S8) underpinning quantitative analyses. System reliability was further validated via solubility measurements (Figure S9).

Sequestration performance was quantified using 4,500 mg·L^−1^ NaCl solution (simulating aquifer brine) and 2.0 wt% erucamine dispersion. CO_2_ sequestration capacity (*q*) was determined for both systems across 8–15 MPa (typical subsurface storage depths; Figure 4B). Equilibrium capacities from *q−t* plots (Figure S10A, B) show that elevating pressure from 8 to 15 MPa increases saline water uptake (*q*_0_) from 71.6 to 116.6 g·L^−1^, while erucamine dispersion uptake (*q*_1_) rises from 91.9 to 138.1 g·L^−1^, increased by nearly 20%. Interestingly, the incremental gain from 2.0wt% erucamine (∆*q* = *q*_1_−*q*_0_) remains consistently ∼20 g·L^−1^ across the pressure range examined.

At constant 10 MPa CO_2_ pressure, increasing erucamine concentration from 1.0 to 5.0 wt% boosts *q* from 110.5 to 170.2 g·L^−1^. Unlike pressure, ∆*q* exhibits pronounced concentration dependence (Figure 4C, Figure S10C), highlighting erucamine content plays a dominant role in modulating sequestration efficiency. This trend is corroborated by observable phase transitions upon CO_2_ exposure (Figure 4D, Movie S4): at higher concentrations, dispersions undergo CO_2_‐triggered transformation into viscoelastic hydrogels, underscoring the role of the material in augmenting CO_2_ retention. A linear correlation between *q* and the resulting hydrogel viscosity (*η*) (Figure 4E) reinforces that CO_2_‐induced viscosity enhancement directly contributes to improved carbon entrapment.

The enhanced CO_2_ sequestration rate (ECSR) displays distinct pressure‐ and concentration‐ dependent behaviors. As pressure increases, ECSR follows a biphasic trend—declining initially before stabilizing near 19.5% (Figure 4F). In contrast, increasing erucamine concentration from 1.0 to 5.0 wt% at 10 MPa drives ECSR from 9.9% to 75.3% (Figure 4G). These trends are mechanistically rationalized in Figure 4H: at fixed erucamine concentration, higher CO_2_ pressure increases CO_2_ availability, shifting the dissolution equilibrium (CO_2_ + H_2_O ⇌ H_2_CO_3_) forward. This proportionally elevates both *q*_0_ and *q*_1_ (Figure 4B), reducing relative enhancement. Conversely, under constant pressure (fixed *q*_0_), higher erucamine concentration promotes protonation, increasing the abundance of erucamine‐H^+^. Here, after the erucamine molecule is protonated, one end is positively charged and exhibits a hydrophilic state, and the long chain with C_22_ tail at one end has a strong hydrophobic effect. This facilitates self‐assembly of WLMs into more extensive gel networks (elevated *q*_1_), physically entrapping additional CO_2_ to boost both ∆*q* and ECSR. These findings delineate a dual mechanism: pressure primarily governs CO_2_ solubility, while erucamine concentration dictates ultimate sequestration capacity through gel network development.

Notably, this work offers direct assessment of material‐mediated CO_2_ sequestration under supercritical conditions (Figure 4I), extending beyond the scope of typical prior studies. Furthermore, comparisons with existing materials confirm that erucamine‐based, CO_2_‐triggered hydrogel systems offer effective gas entrapment (Figure 4J). Unlike previously reported materials, CO_2_‐responsive hydrogels represent a novel class of sequestration medium capable of achieving stable carbon storage under realistic saline aquifer conditions, highlighting their promise for practical carbon capture and storage applications.

### 2.5 Enhanced CO_2_ Sequestration With Tailored DESs

**FIGURE 5:**
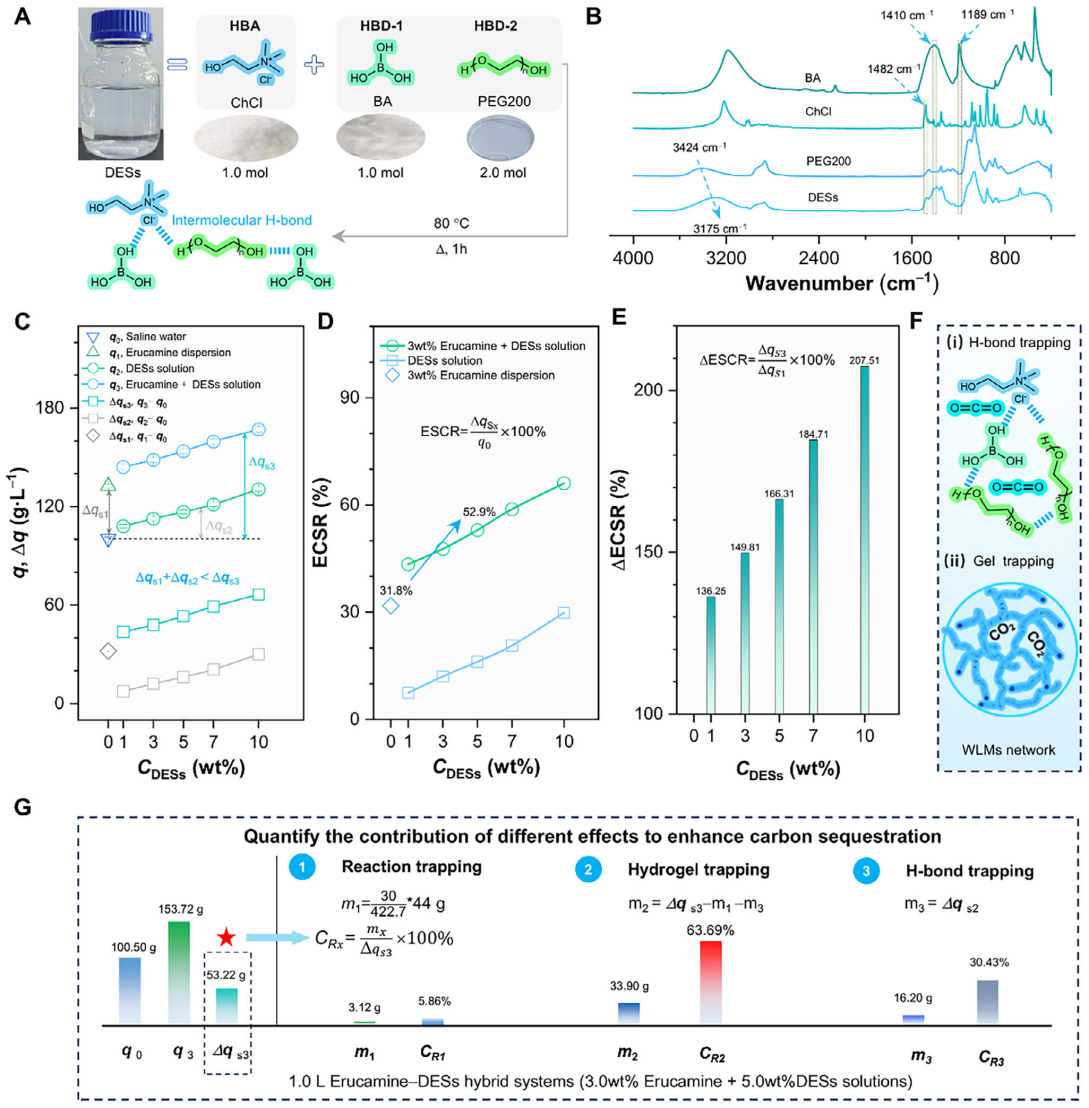
Composition, structure and CO_2_ sequestration performance of DESs. (A) Preparation and visual appearance of synthesized DESs. (B) ATR‐FTIR spectra of DES precursors and the resultant mixtures. (C) Comparative CO_2_ uptake capacities of the investigated systems at 45°C and 10 MPa CO_2_. (D) Concentration‐dependent ECSR of each system at 45°C and 10 MPa CO_2_. (E) Influence of DES content on sequestration performance within 3.0 wt% erucamine dispersion at 45°C and 10 MPa CO_2_. (F) Proposed dual‐mechanism framework for enhanced CO_2_ trapping. (G) Quantitative breakdown of individual contributions to the overall enhancement. *q*_0_ presents the carbon sequestration capacity of 4,500 mg·L^−1^ NaCl solution, as a baseline; *q*_3_ is the carbon storage capacity of 3 wt% erucamine + 5 wt% DES solutions, and Δ*q_s_*_3_ = *q*_3−_ *q*_0._

To optimize performance while preserving favorable solubility, ternary hydrophilic DESs (Figure 5A) were formulated using boric acid (BA), choline chloride (ChCl) and polyethylene glycol 200 (PEG200)—the latter selected for its intrinsic CO_2_ affinity [76] to promote adsorption. Attenuated total reflectance–Fourier transform infrared (ATR‐FTIR) spectroscopy monitored structural evolution during DES formation (Figure 5B). In pure BA, characteristic bands appear at 1410 cm^−1^ (B–O) and 1189 cm^−1^ (O–H) [77]. Following DES assembly, the O–H vibration either disappears or redshifts, indicating hydrogen‐bond establishment. Concurrently, the distinct ChCl peak at 1482 cm^−1^ diminishes or merges, while the −OH stretching band of PEG200 shifts from 3424 to 3175 cm^−1^. These spectral changes collectively confirm that DESs formation is driven by an extended hydrogen‐bond network [78].

Incorporating 5.0 wt% DESs into 3.0 wt% erucamine dispersion yielded a visually homogeneous system. Notably, the 3.0 wt % erucamine concentration was selected for DES‑doping experiments because it provides an optimal balance between injectability (low initial viscosity) and robust CO_2_‑responsive gelation. This condition enables unambiguous observation of synergistic enhancement while avoiding performance saturation. Rheological analysis showed DESs addition elevated initial viscosity from 3.9 to 5.4 mPa·s; upon CO_2_ exposure, viscosity surged to 113.2 mPa·s (Figure S11), confirming retained hydrogel‐forming capability and favorable sequestration characteristics.

To quantify DESs integration effects, CO_2_ uptake was evaluated for pure DESs and erucamine–DESs hybrid systems across 1.0–10.0 wt% DES concentrations. Pure DESs exhibited concentration‐dependent capture capacity (*q*), rising from 107.9 to 130.5 g·L^−1^. In contrast, the hybrid systems achieved substantially enhanced values (144.1–166.9 g·L^−1^; Figure 5C, Figure S12).

The ECSR of the hybrid formulations consistently surpassed individual constituents, evidencing a synergistic effect (Δ*q*_s3_>Δ*q*_s1_ + Δ*q*_s2_; Figure 5D). Quantified by the synergistic coefficient ΔECSR, this cooperation increased from 136.2% to 207.5% with rising DES concentration (Figure 5E). These observations support two key conclusions: (i) DESs significantly augment erucamine‐mediated CO_2_ sequestration, and (ii) enhancement operates via a dual mechanism combining hydrogen‐bond trapping and hydrogel entrapment (Figure 5F).

Deconvolution of total capacity increase (Δ*q*_s3_) in a representative 1.0 L hybrid system elucidated the relative contributions. For this quantitative deconvolution analysis, the solubility‑trapping baseline corresponds to the CO_2_ uptake capacity of pure brine (4500 mg·L^−1^ NaCl solution) in the absence of erucamine and DES additives. All enhancement magnitudes are computed by subtracting this baseline value. Full calculation workflows are illustrated in Figure 5G, enabling individual contribution quantification for each trapping mechanism. Hydrogel trapping dominated, accounting for 63.69% of enhancement (*m*_2_/Δ*q*_s3_). Hydrogen‐bond trapping contributed 30.43% (*m*_3_/Δ*q*_s3_), while reaction consumption represented a minor fraction (5.86%, equivalent to 3.12 g CO_2_; Figure 5G). Thus, hydrogel formation emerges as the primary driver of cooperative sequestration enhancement.

### 2.6 Injection Capacity and Long‐Term Stability

**FIGURE 6:**
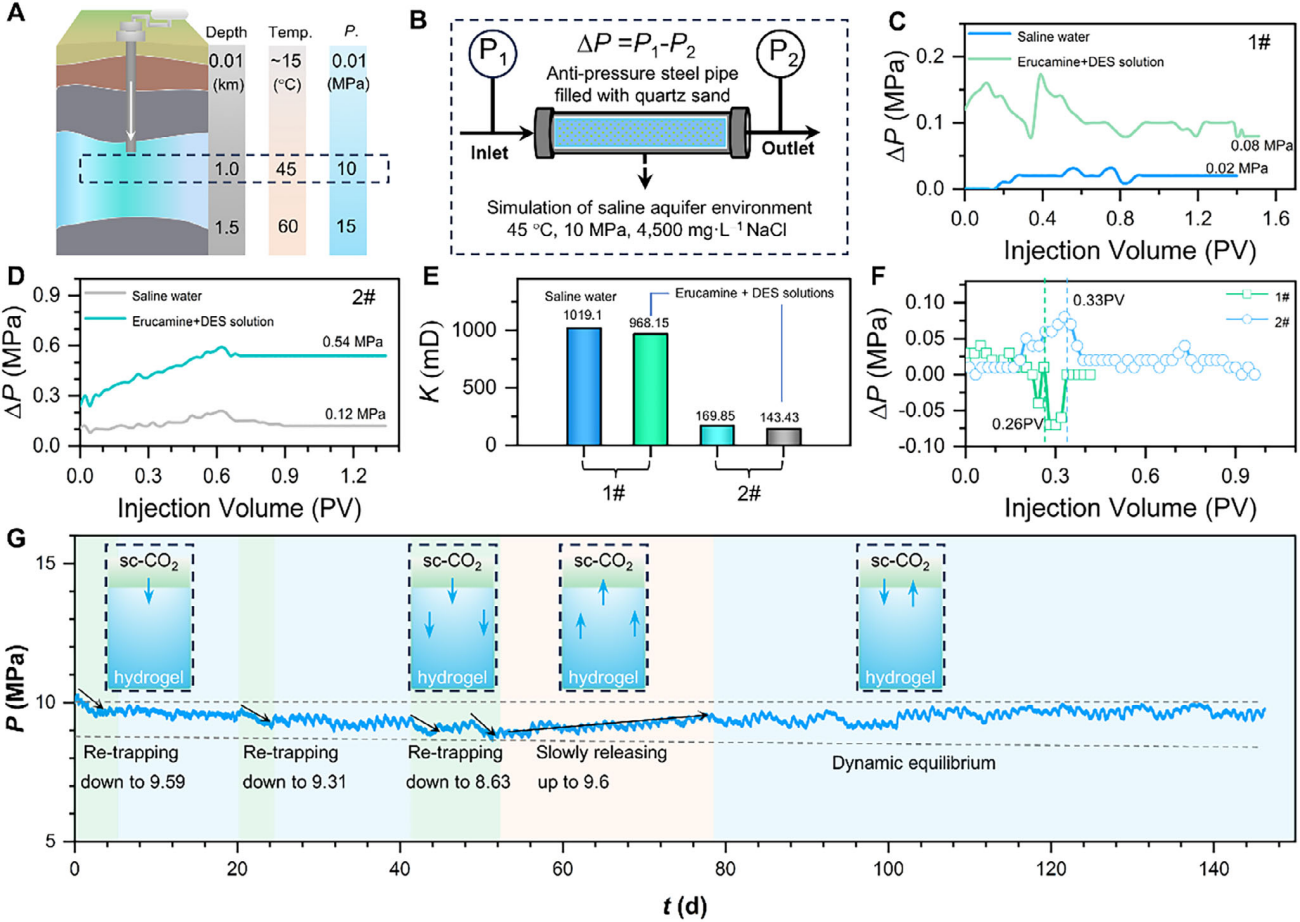
Validation of injectability and long‐term stability. (A) Schematic of a typical saline aquifer environment. (B) Laboratory setup simulating saline aquifer conditions using a sand‐packed column. (C) Injection behavior of fluids in a high‐permeability saline aquifer (1#) at 45°C and 10 MPa. (D) Injection behavior in a medium‐permeability aquifer (2#) at 45°C and 10 MPa. (E) Comparison of effective permeability for different injected fluids at 45°C and 10 MPa. (F) Differential pressure evolution during CO_2_ injection into aquifers of varying permeability at 45°C and 10 MPa. (G) Long‐term stability of in situ formed CO_2_‐induced hydrogels for carbon sequestration at 45°C and 10 MPa.

Injectability of the erucamine and DESs mixed solution is a fundamental prerequisite for the practical deployment of in situ CO_2_‐induced hydrogel systems. To assess this critical characteristic, a laboratory‐scale saline aquifer simulation environment (Figure 6A) was established. The experimental setup comprised a high‐pressure steel column packed with quartz sand and saline water, integrated with temperature and pressure controls to replicate representative subsurface conditions (Figure 6B). Injectability was evaluated by monitoring the differential pressure (ΔP = P_1_−P_2_) across the inlet and outlet during fluid injection.

In the high‐permeability simulated formation (pore volume, PV = 52.7 mL; porosity, *ϕ*= 35.7%), stabilized injection differential pressure was 0.02 MPa for brine and 0.08 MPa for the erucamine and DESs mixed solution (Figure 6C). Under medium‐permeability conditions (PV = 48.1 mL; *ϕ *= 32.6%), corresponding values were 0.12 MPa (saline water) and 0.54 MPa (Erucamine + DESs solution; Figure 6D). These modest pressure increases confirm favorable injectability of the gel‐forming fluid across a range of permeabilities.

Permeability analyses further corroborate these findings, showing only marginal differences between brine and the erucamine and DESs mixed solution (Figure 6E). This suggests that the hydrogel precursor can penetrate porous media without inducing significant flow obstruction. Following injection of the erucamine and DESs mixed solution, CO_2_ was introduced into the system: breakthrough occurred after 0.26 PV in high‐permeability setup, while reduced permeability delayed breakthrough to 0.33 PV (Figure 6F). These observations imply moderate‐to‐low permeability formations may offer more favorable conditions for retarding gas channeling and improving containment efficiency.

Long‐term sequestration stability was evaluated over a continuous monitoring period of 146 days at 45°C. No CO_2_ leakage was detected inside the closed HTHP reactor across the 146‑day monitoring window, confirming favorable storage stability. Throughout this duration, system pressure remained consistently below the predefined safety threshold of 10 MPa, indicating no detectable CO_2_ leakage (Figure 6G). Pressure profile analysis further revealed the hydrogel enables sustained CO_2_ reabsorption (pressure down to 8.63 MPa), followed by gradual release (pressure up to 9.6 MPa), ultimately reaching dynamic equilibrium within the confined environment.

In short, combined assessments of injectability and extended stability substantiate the technical feasibility of the in situ CO_2_‐induced hydrogel strategy. These findings underscore its potential as a robust, viable approach for long‐term carbon sequestration in saline aquifer settings.

### 2.7 Mechanism Underlying Enhanced Carbon Sequestration

Prior observations establish that erucamine enhances carbon sequestration in saline aquifers, an effect further amplified by the DESs incorporation. To elucidate the mechanistic roles of both components, three complementary characterization techniques were employed.

**FIGURE 7:**
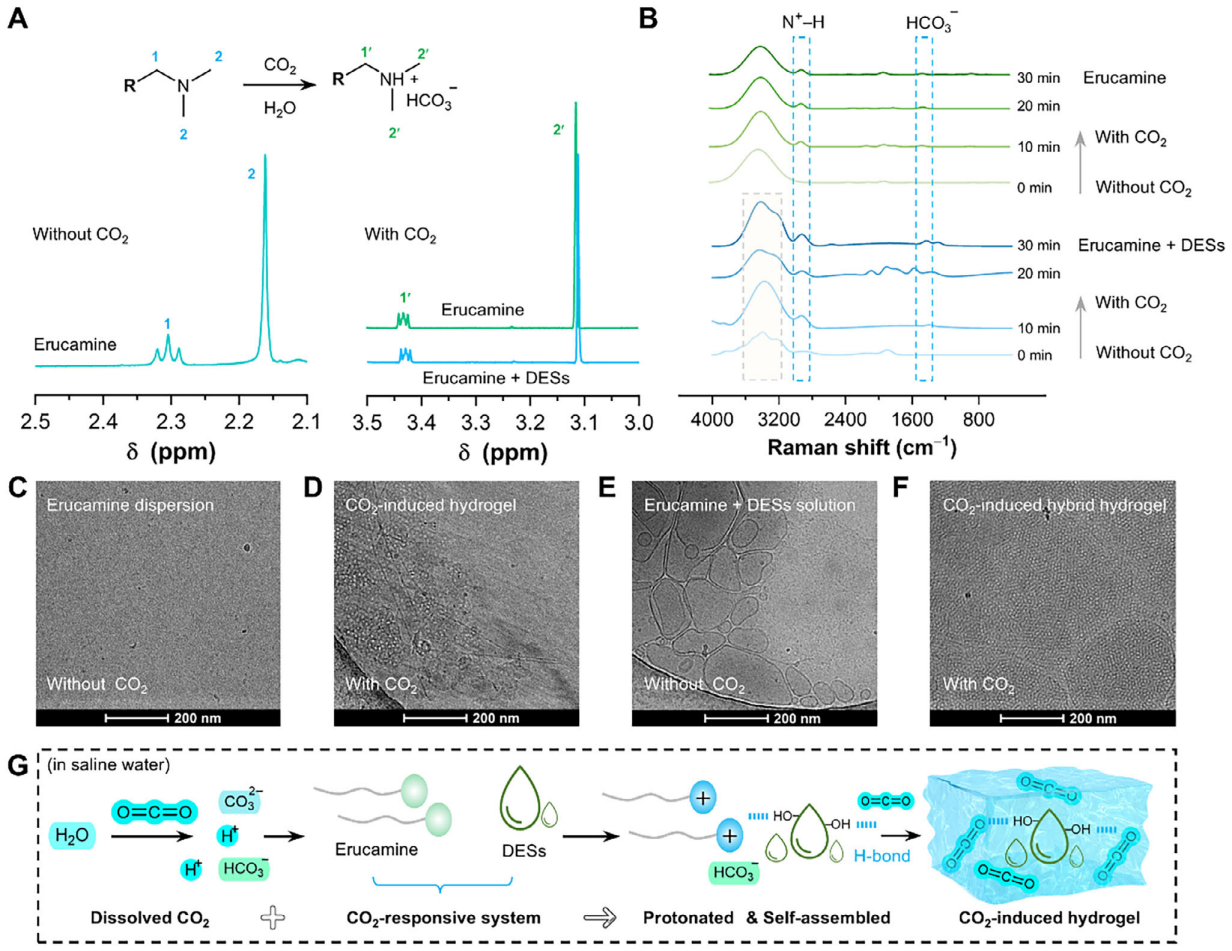
Mechanism of enhanced carbon sequestration via erucamine and DESs. (A) Protonation of erucamine (top) and corresponding ^1^H NMR spectra (bottom, in CDCl_3_) before CO_2_ exposure (cyan) and after protonation in D_2_O (green). Spectrum of the protonated mixture in D_2_O (blue) with labelled chemical shifts (1, 1′, 2, and 2′). (B) In situ Raman spectra of 3.0 wt% erucamine (green) and erucamine‑DESs mixture (blue) during CO_2_ treatment (characteristic peaks marked with dotted boxes) at 45°C. Cryo‐TEM micrographs of 3.0 wt% erucamine in 4,500 mg·L^−1^ NaCl. (C) Prior to CO_2_ introduction and (D) following exposure to 10 MPa CO_2_ at 45°C. Cryo‐TEM image of a mixed solution containing 3.0 wt% erucamine and 5.0 wt% DESs (4,500 mg·L^−1^ NaCl). (E) Before CO_2_ treatment and (F) after treatment with 10 MPa CO_2_ at 45°C. (G) Schematic depiction of the structural evolution in the erucamine ‐DESs hybrid system upon CO_2_ exposure, correlating with improved carbon sequestration.

^1^H NMR spectroscopy monitored chemical changes upon CO_2_ exposure. In the absence of CO_2_, the tertiary amine protons of erucamine resonate at 2.3 ppm (1) and 2.16 ppm (2) (Figure 7A). Upon CO_2_ introduction, these signals shift identically in both pure erucamine and erucamine‐DESs solutions. This consistent downfield migration confirms amine protonation, corroborating the reaction‐based trapping mechanism outlined in Figure 1C. Importantly, DESs do not alter this protonation event, suggesting an auxiliary rather than direct chemical role.

In situ Raman spectroscopy further probed the enhancement mechanism. Prior to CO_2_ treatment, erucamine dispersions lacked characteristic signatures of HCO_3_^−^ or N^+^–H (Figure 7B). Following CO_2_ exposure, the emergence of the N^+^–H vibrational mode confirmed amine protonation—a response replicated in the DESs‐containing mixture. Crucially, the –OH stretching band centered at 3380 cm^−1^ exhibited marked broadening and superposition, indicative of new intermolecular interactions. These spectral features point to hydrogen bonding between dissolved CO_2_ species and DES constituents.

Ex situ cryo‐TEM visualized microstructural evolution during CO_2_‐induced phase transitions (Figure 7C–F). The pristine erucamine dispersion contained spherical micelles (Figure 7C), which transformed into interconnected WLMs (Figure 7D) via supramolecular assembly upon CO_2_ exposure. In contrast, the initial erucamine‐DESs mixture exhibited polydisperse multilamellar vesicles (Figure 7E). After CO_2_ treatment, these vesicles rearranged into an ordered hexagonal liquid crystalline phase (Figure 7F). This structural reorganization stems from hydrophilic DESs modulating supramolecular interactions, promoting a more ordered architecture. The proposed transformation pathway for the hybrid system is schematically summarized in Figure 7G.

In summary, enhanced CO_2_ sequestration capacity originates from CO_2_‐triggered supramolecular self‐assembly of erucamine, reinforced by robust intermolecular hydrogen bonding facilitated by DESs—synergistically amplifying carbon trapping efficiency.

## 3 Conclusions and Outlook

This study introduces a novel strategy for boosting carbon sequestration in saline formations via the synergistic integration of CO_2_‐responsive hydrogel and DESs. CO_2_‐triggered hydrogels formed from the erucamine self‐assembly as tailored sequestration media, amplifying the inherent storage potential of the aquifer matrix—an advancement with substantial industrial relevance. Central to this mechanism is the sol–gel transition of a CO_2_‐responsive materials(erucamine), which generates stable hydrogels optimized for geological confinement, while DESs impart additional non‐covalent trapping interactions to synergistically enhance both subsurface carbon storage capacity and efficiency.

A key outcome of this work lies is the development of a direct quantification framework for evaluating liquid‐phase storage media, providing a robust tool for performance benchmarking. Experimental results confirm that the integrated material system effectively enhances CO_2_ sequestration. Injection tests under simulated reservoir conditions demonstrate favorable injectivity, satisfying a critical prerequisite for field‐scale deployment. For practical field‐scale deployment in saline aquifers, both reservoir heterogeneity and on‐site operational uncertainties need to be fully considered. The material developed in this work can selectively form hydrogels in high‐permeability channeling pathways and thus inhibit CO_2_ gas breakthrough. To adapt to actual field conditions and improve sequestration performance, a segmented injection strategy analogous to the WAG technique is proposed. This strategy can effectively suppress gas channeling and enhance CO_2_ storage capacity, while the slug injection design minimizes the adverse dilution effect of formation water to guarantee stable gelation behavior. Furthermore, downhole real‐time monitoring of in‐situ material responses can provide solid guidance for future mechanism research and field operation optimization.

Although focused on CO_2_‐responsive hydrogel–DESs hybrids, the underlying principles are transferable to other advanced material systems, such as stimuli‐responsive polymers and microporous frameworks. In conclusion, these findings lay a theoretical and practical foundation for overcoming carbon storage limitations in saline aquifers. The proposed strategy holds significant promise for advancing CCUS technologies, contributing to sustainable development through safer, more efficient geological carbon sequestration.

Future investigations could extend to optimizing material formulations for extreme reservoir conditions (e.g., high salinity, elevated temperatures) and scaling up via pilot‐scale field trials. Expanding the scope to other anthropogenic greenhouse gases or integrating with renewable energy‐driven CO_2_ capture processes may further broaden the strategy's environmental impact, aligning with global net‐zero emission objectives.

## 4 Materials and Methods

### 4.1 Materials

Sodium chloride (NaCl, ≥99%), choline chloride (ChCl, 98%), and boric acid (BA, 99%) were procured from Adamas (Shanghai, China). Polyethylene glycol (PEG200) was acquired from McLean Biochemical Technology (Shanghai, China). Quartz sand with particle size fractions of 40–70, 110–160, and 160–200 mesh was supplied by Tianyi Technology (Wuhan, China). High‐purity CO_2_ and N_2_ (both ≥ 99.99%) were provided by Tianyuan Gas (Chengdu, China). Ultrapure deionized water (resistivity: 18.25 MΩ·cm), produced by a CDUPT‐III water purification system (Chengdu Yuechun Technology, China), was used for preparing all aqueous solutions.

### 4.2 Preparation of Erucamine Dispersions

N‐erucamidopropyl‐N,N‐dimethylamine (erucamine) was synthesized according to a previously reported protocol [60]. Its chemical structure and molecular weight were verified by nuclear magnetic resonance (NMR) and mass spectrometry (Figures S13 and S14), consistent with theoretical values. A saline solution containing 4,500 mg·L^−1^ NaCl was prepared by dissolving NaCl powder in ultrapure water and served as the solvent for erucamine, following an established protocol [50]. Precisely weighed dry erucamine powder was dissolved in this brine via heating in a 60°C water bath. The turbid solution was rendered transparent by CO_2_ sparging until complete dissolution; subsequent N_2_ purging converted the clear solution into a low‐viscosity, water‐like fluid.

### 4.3 Formulation and Characterization of the DESs

DESs were formulated by homogenizing ChCl, BA, and PEG200 at a molar ratio of 1:1:2 and heating the mixture at 80°C for one hour. The resultant homogeneous transparent liquid was used without further purification. Solvent structure and intermolecular interactions were characterized by Attenuated Total Reflectance Fourier Transform Infrared (ATR‐FTIR) spectroscopy over a spectral range of 4000–400 cm^−1^, with a resolution of 0.5 cm^−1^ and a scanning rate of 1 cm^−1^.

### 4.4 Preparation of Erucamine‐DES Mixtures

Mixed systems contained 3.0 wt% erucamine, 1–10 wt% DESs, and 87–96 wt% brine (4,500 mg·L^−1^). As a representative example: 92.0 g of brine was combined with 3.0 g of erucamine powder under agitation until fully dissolved. Subsequently, 5.0 g of DESs was incorporated and mixed thoroughly; continuous N_2_ sparging for 30 min eliminated dissolved air, yielding a uniform solution with 3.0 wt% erucamine and 5.0 wt% DESs.

### 4.5 In Situ Visualization of CO_2_‐Induced Sol–Gel Transition

CO_2_‐triggered sol–gel transitions were visualized at 10 MPa and 45°C using two platforms: a commercial HTHP cell (DSA100HP1750, Germany; Figure S1A) equipped with an integrated dynamic recording system, and a custom‐built HTHP visualization setup (Figure S1B) designed for real‐time data acquisition. Detailed experimental methods (verification and judgment) and parameters were provided in Supporting Information Text S1.

### 4.6 Rheological Measurements Under Simulated Reservoir Conditions

Rheological properties of the erucamine dispersions were evaluated using an HTHP rotational rheometer (MCR302, Anton‐Paar, Austria; Figure S2) to replicate saline aquifer storage conditions. All measurements were conducted with the CC33.2 measuring system; specific test parameters were detailed in Supporting Information Text S2.

### 4.7 Assessment of CO_2_ Sequestration Capacity

CO_2_ sequestration performance of brine, erucamine dispersions, DESs solutions, and their combinations was assessed using a custom‐designed HTHP reaction system with real‐time monitoring (Figure S6 and Text S6). System integrity was verified via leak testing prior to each experiment (Figure S7, Text S7). Comprehensive experimental details were provided in Text S6. Total CO_2_ sequestration capacity (*q*) was calculated using Equations (1) – (4):

(1) $$q=\frac{m_{1}-m_{2}-m_{3}}{V_{0}}$$

(2) $$m_{1}=\int_{0}^{t}Q_{\mathrm{in}}dt$$

(3) $$m_{2}=\int_{0}^{t}Q_{\mathrm{out}}dt$$

(4) $$m_{3}=\rho V_{1}$$

where, *m*_1_ and *m*_2_ represent the cumulative mass of CO_2_ entering and exiting the system, respectively; *m*_3_ denotes residual CO_2_ mass in the reactor (derived from temperature‐ and pressure‐dependent density, Figure S8); *V*_0_ was the sample volume; *Q*_in_ and *Q*_out_ refer to inlet and outlet mass flow rates; *t* indicates injection duration; and *V*_1_, stands for the fixed residual reactor volume (50 mL).

### 4.8 Simulated Injection Into Saline Aquifers

Injection experiments were conducted to evaluate the permeability and flow characteristics of the sample solutions—key indicators of practical applicability. CO_2_ sequestration within the column was further evaluated by injecting CO_2_ into the sample‐saturated porous medium, with sequestration extent inferred from pressure decay. A sand‐packed column (2.5 cm diameter × 30 cm length) served as a surrogate saline aquifer. Quartz sand of varying mesh sizes (40–70, 110–160, and 160–200) was packed in defined proportions to create two columns with distinct permeabilities, following standard sandstone packing protocols. The sand was intrinsically water‑wet, which was representative of most saline aquifers. Pore volume (PV) and porosity (*ϕ*) were determined using Equations (5) and (6):

(5) $$PV=\frac{w_{2}-w_{1}}{\rho_{\mathrm{brine}}}$$

(6) $$\phi =\frac{PV}{V_{0}}\times 100\%$$

where, *ρ*_brine_ was the density of the 4500 mg·L^−1^ NaCl solution (g·cm^−3^); *V*_0_ denotes the bulk volume of the column (cm^3^); and *w*_1_ and *w*_2_ were the mass of the column before and after saturation with saline water (g), respectively.

Brine or sample solution was infused at 2.0 mL·min^−1^, with continuous recording of inlet (*P*_1_) and outlet (*P*_2_) pressures. Permeability (*K*, µm^2^ or Darcy) was calculated via Darcy's law (Equation 7):

(7) $$K=\frac{Q\eta L}{10A\Delta P}$$

where, *Q* was the volumetric flow rate (cm^3^·s^−1^); *η *indicates fluid viscosity (mPa·s); *L* represents the column length (cm); Δ*P* stands for the pressure drop across the column (MPa); and *A* refers to the cross‐sectional area (cm^2^).

### 4.9 Long‐Term Thermal Stability Tracking

Long‐term stability of the hybrid hydrogels for CO_2_ sequestration was monitored using the HTHP reaction system, comprising injection, monitoring, and backpressure control units. First, 200 mL of erucamine and DESs mixed solution was injected the HTHP system (maintained at 45°C) via an ISCO pump at 2.0 mL·min^−1^. CO_2_ was then injected at 0.5 mL·min^−1^ until the system reached 10 MPa backpressure. Pressure was continuously monitored under simulated reservoir conditions (45°C, 10 MPa) for extended periods. Stable pressure indicated effective CO_2_ retention, while a rise suggested CO_2_ release and a decline implied incomplete sequestration.

### 4.10 ^1^H NMR Spectroscopy

Erucamine's molecular structure was characterized by ^1^H NMR spectroscopy in deuterated chloroform (CDCl_3_). Additional spectra were acquired in deuterated water (D_2_O) for pure erucamine dispersions, CO_2_‐saturated erucamine dispersions, and erucamine‐DESs mixtures under CO_2_ atmosphere. All spectra were collected at 25°C on an AVANCE II 600 MHz spectrometer (Bruker, Germany).

### 4.11 In Situ Raman Spectroscopy

In situ Raman spectra were collected using a confocal microscope Raman system (HORIBA, France) with a 532 nm excitation laser. Frequency calibration was performed prior to each measurement using a silicon wafer reference. Spectra were acquired at 45°C under a continuous CO_2_ atmosphere at 10‐min intervals over a spectral range of 4000–400 cm^−1^, with a resolution of 0.5 cm^−1^ and a scanning rate of 1 cm^−1^.

### 4.12 Cryo‐TEM

Cryo‐transmission electron microscopy (Cryo‐TEM) was employed to observe microstructures of four distinct systems: pristine erucamine dispersion, erucamine–DESs hybrid solution, and their CO_2_‐sequestrated counterparts (10 MPa CO_2_, 45°C). Samples were prepared per a reported protocol [79]: 2.0 µL of solution was applied to a holey carbon‐coated TEM grid, blotted to form a thin film, and rapidly plunge‐frozen in liquid ethane (−180°C) cooled by liquid nitrogen. Grids were stored at ≈ −196°C until imaging. Morphological comparisons were performed at identical magnifications to elucidate structural transformations.

## Author Contributions

**Fuchuan Liu**: methodology, investigation, formal analysis, data curation, Writing – original draft. **Xinjie Luo**: methodology, investigation, formal analysis, visualization. **Dianguo Wu**: validation, investigation. **Xuezhi Zhao**: visualization, validation, resources. **Yan Zhang**: validation, methodology, project administration, resources, writing – review and editing, funding acquisition. **Yujun Feng**: conceptualization, funding acquisition, supervision, writing – review and editing.

## Conflicts of Interest

The authors declare no conflicts of interest.

## Supporting information




**Supporting File 1**: advs77958‐sup‐0001‐SuppMat.docx.

**Supporting File 2**: advs77958‐sup‐0002‐MovieS1.mp4.

**Supporting File 3**: advs77958‐sup‐0003‐MovieS2.mp4.

**Supporting File 4**: advs77958‐sup‐0004‐MovieS3.mp4.

**Supporting File 5**: advs77958‐sup‐0005‐MovieS4.mp4.

## Data Availability

The data that support the findings of this study are available on request from the corresponding author. The data are not publicly available due to privacy or ethical restrictions.
