# Supplementary material for: In Situ CO2‐Induced Hydrogels for Enhancing Carbon Sequestration in Saline Aquifers
Source: Adv Sci (Weinh). 2026 Sep 27:e77958. Online ahead of print. doi: 10.1002/advs.77958 (PMC13616250; doi:10.1002/advs.77958)
Supplement: Supplementary file 1 [file ADVS-9999-e77958-s005.docx]

Supporting Information

In Situ CO_2_-induced Hydrogels for Enhancing Carbon Sequestration in Saline Aquifers

Fuchuan Liu, Xinjie Luo, Dianguo Wu, Xuezhi Zhao, Yan Zhang,^*^ and Yujun Feng^*^

Polymer Research Institute, State Key Laboratory of Advanced Polymer Materials, Sichuan University, Chengdu 610065, People’s Republic of China

*Corresponding Authors: Yan Zhang, Yujun Feng

Email: y.zhang@scu.edu.cn (Y. Zhang), yjfeng@scu.edu.cn (Y. Feng).

Table of contents

Text S1 to S13

Figs. S1 to S14

Tables S1

Movies S1 to S4

References 1 to 19

**S1 |** **Commercial and Custom-built Visualization Systems and Experimental Parameters**

The high-pressure drop shape analyzer (DSA100HP1750, Germany) utilized in this study is depicted in Figure S1A. This commercial instrument enables precise measurement of contact angles, surface tensions and interfacial tensions under extreme conditions (up to 1750 bar and 250 °C). Its sealable visualization chamber facilitated dynamic monitoring of phase behavior throughout the experiments. In this work, this apparatus was utilized for in situ monitoring of hydrogel formation. The detailed experimental procedures are described as follows: (1) Inspect and clean the visualization window and reaction cell. (2) Assemble the visualization cell, followed by gas injection to test the air‑tightness of the cell. (3) After the air‑tightness test, purge residual gas from the cell, and inject a fixed volume of erucamine dispersion from the bottom inlet of the cell. (4) Adjust the camera lens to achieve clear observation of the dispersion state inside the cell. (5) Inject a small volume of dispersion from the cell bottom again and record the liquid‑level change (control group). Subsequently, inject CO_2_ up to 0.3 MPa via the gas inlet and record the evolution of the dispersion (control group). (6) Continuously inject CO_2_ until the cell pressure reaches 10 MPa. Under 10 MPa CO_2_, record the phase‑transition process by video recording. (7) Upon completion of the phase transition, inject additional liquid and gas into the cell, and record the internal morphological changes. (8) After the experiment, depressurize the system, remove the sample, clean the cell thoroughly, and restore all components to their initial state.

**FIGURE S1** | Two visualization setups. (A) Commercial apparatus showing the overall assembly (left) and a close-up of the observation window (right). (B) Custom-built experimental flow visualization system, featuring the complete setup alongside a detailed view of the observation chamber.

For real-time color imaging with simultaneous automated recording of temperature and pressure, a custom high-pressure, high-temperature (HTHP) visualization platform (Figure S1B) was constructed. This integrated system incorporates calibrated sensors paired with digital data loggers for continuous pressure and temperature tracking, while optical access *via* a quartz window allows high-resolution (2 K) imaging using an externally mounted CCD camera.

In each experimental run, aqueous erucamine dispersion were doped with bromothymol blue (BTB, pH 6.0–7.6) to enable concurrent visual observation of phase behavior and indirect confirmation of CO_2_ encapsulation. Specifically, 10.0 mL of a 5.0 wt% erucamine dispersion was loaded into a dual-window transparent HTHP cell, equilibrated at 45 °C, and subsequently pressurized with CO_2_ to 10 MPa. The entire process was continuously recorded using an 800TVL CCD camera until gelation completion. Controlled depressurization was similarly documented, with subsequent video analysis yielding insights into transition kinetics and sequestration dynamics.

**S2 |** **HTHP Rheometer and Rheological Characterization**

An HTHP rheological testing system was integrated based on a Physica MCR302 rotary rheometer (Anton-Paar, Austria; Figure S2). The apparatus comprises four subsystems: (1) Gas inlet and pressurization, regulating pressure within the measurement cell *via* a gas cylinder and booster; (2) Sample loading and testing, utilizing a hermetically sealed CC33.2 geometry; (3) Temperature control, combining internal heating (C-PTD200) with external cooling for precise thermal management; and (4) Data acquisition, managed by Anton-Paar proprietary software for real-time monitoring and recording. Compressed air from an external compressor powers the mechanical functions of the rheometer.

**FIGURE S2** | Configuration of the rotational rheometer equipped with a CC33.2 measuring system.

Rheological behaviors of erucamine dispersion were evaluated using this HTHP system to simulate storage conditions relevant to saline aquifer, with all tests employing the CC33.2 geometry. Experimental parameters varied as detailed below:

*Shear viscosity and injectability*. Injectability was assessed by measuring shear viscosity at a constant shear rate of 100 s^−1^, mimicking pump-controlled subsurface delivery. The system was pressurized with N_2_ and heated from 20 °C to 45 °C at 3 °C·min^−1^. To simulate post-injection conditions, N_2_ was replaced with CO_2_ (10 MPa, 45 °C), and viscosity was remeasured at the same shear rate.

*Rheological behavior*. Flow properties of CO_2_-induced hydrogels under reservoir conditions (45 °C, 10 MPa CO_2_) were characterized through dynamic rheological tests: a cyclic logarithmic shear-rate sweep (0.001–1,000 s^−1^) probed behavior across different deformation regimes; time-sweep oscillatory tests monitored elastic (*G*′) and viscous (*G*″) moduli to identify the gelation point; and oscillatory-shear-oscillatory tests investigated structural recovery —oscillatory phases (1% strain, 1 Hz) quantified viscoelastic properties, while an intermittent shear phase (100 s^−1^) assessed viscosity during breakdown.

*Steady-state rheological properties*. Steady-state shear tests determined the viscosity of CO_2_-induced hydrogels as a function of pressure and concentration (shear rate: 100 s^−1^; temperature: 45 °C). Samples were equilibrated at target temperature for 20 min, subjected to a pre-shear step (100 s^−1^ for 3 min) to ensure consistent initial conditions, and rested for 10 min to minimize transient effects.

**S3 |** **Viscosity Characteristics of Erucamine Dispersion**

Viscosity profiles of erucamine dispersion at various concentrations were measured throughout the simulated injection process (Figure S3). All dispersion exhibited temperature-dependent viscosity reduction; however, following CO_2_ injection, viscosity increased markedly after a defined reaction period, with higher-concentration formulations achieving higher plateau values. These observations confirm CO_2_ triggers structural reorganization within the dispersion, leading to a macroscopic rheological shift, specifically, a measurable viscosity enhancement.

**FIGURE S3** | Rheological behavior of erucamine dispersion during sequential injection and CO_2_ sequestration stages. Viscosity evolution at a shear rate of 100 s^−1^ under (i) simulated erucamine injection (20–45 °C) and (ii) subsequent CO_2_ sequestration. The accompanying temperature profile reflects the thermal transition from the surface to saline aquifer conditions.

**S4 |** **Rheological Behavior of CO_2_-Treated Erucamine Dispersion**

Flow curves for CO_2_-treated erucamine dispersion of varying are displayed in Figure S4. The 1.0 wt% solution retained fluid characteristics, evidenced by a measurable zero-shear viscosity plateau. In contrast, dispersion at concentrations of 2.0 wt% and above lacked such a plateau, consistent with gel formation under quiescent conditions [1].

**FIGURE S4** | CO_2_-induced flow behavior of erucamine dispersion (1.0–5.0 wt%) upon exposure to 10 MPa CO_2_ at 45 °C.

**S5 |** **Steady-Shear Viscosity Profiles of CO_2_-treated Erucamine Dispersion**

Steady-shear viscosity of CO_2_-exposed erucamine dispersion was investigated as a function of pressure and concentration (Figure S5). Applied pressure exerted a negligible influence on viscosity, while the solution concentration emerged as the predominant determinant of the rheological response.

**FIGURE S5** | Effects of pressure and concentration on viscosity. (A) Time-dependent steady-shear viscosity of a 2.0 wt% erucamine solution exposed to 8–15 MPa CO_2_ at 45 °C and 100 s^−1^. Data for each pressure are shown separately to highlight minimal pressure dependence. (B) Time-dependent steady-shear viscosity of erucamine hydrogels (1.0–5.0 wt%) under 10 MPa CO_2_ at 45 °C and 100 s^−1^.

**S6 |** **Details of the CO_2_ Sequestration Testing Apparatus and Experimental Parameters**

Building upon previously reported HTHP CO_2_ solubility testing systems [2-4], a reconfigurable apparatus was developed for simultaneous solubility measurement and sequestration capacity evaluation. The system integrates five modules (Figure S6): a gas pressurization unit, a CO_2_ injection and delivery unit, an HTHP reactor for sample containment, a pressure and temperature regulation unit, and a gas‒liquid separation unit.

**FIGURE S6 |** Schematic diagram of the experimental configuration employed for CO_2_ sequestration.

A key innovation of this apparatus lies in precision Coriolis mass flowmeters at both the inlet and outlet of the HTHP reactor: the inlet flowmeter (ACU 20FE-LM; max. flow: 300 g⋅h^−1^; accuracy: ±0.01%) directly measures supercritical and liquid CO_2_ mass flow, while the outlet flowmeter (ACU 20FE-G; max. flow: 300 g⋅h^−1^; accuracy: ±0.01%) monitors gaseous CO_2_. Flow data are continuously recorded via a digital acquisition system. The reactor (250 mL capacity) features the following specifications:

• Pressure range: 0–50 MPa

• Temperature range: 25–180 °C

• Material: Hastelloy alloy (selected for corrosion resistance and chemical inertness)

Real-time monitoring is achieved through an integrated pressure transducer (accuracy: ±1.0%) and a temperature probe (accuracy: ±1.0%), with all sensor outputs logged synchronously.

For each experiment, 200 mL of test fluid was loaded into the reactor, equilibrated at 45 °C, and liquid/supercritical CO_2_ was introduced at 2.0 mL⋅min^−1^ via a syringe pump. An outlet regulator-maintained backpressure to simulate aquifer conditions. Once backpressure threshold was exceeded, effluent CO_2_ passed through the gas–liquid separator and the outlet mass flowmeter. Dynamic sequestration equilibrium was assumed when inlet and outlet flow rates stabilized within experimental uncertainty, at which point CO_2_ injection ceased. Pressure and mass flow data were registered at 5-second intervals using a high-precision acquisition system (apparatus uncertainty: ±1%).

**S7 |** **Airtightness Inspection of the CO_2_ Sequestration Device**

Prior to measurements, the airtightness of the system was rigorously verified. CO_2_ was introduced into the sealed circulatory system and pressurized, with pressure stability monitored under isothermal conditions for a minimum of 45 min. If pressure fluctuations exceeded an acceptable threshold (0.5%), the apparatus was inspected for leaks until the variation fell below this limit. As illustrated in Figure S7, reactor pressure (blue hollow circles) remained stable post-pressurization, confirming the airtight integrity of the system. Additionally, inlet (black) and outlet (green) flow rates reached equilibrium with no discernible deviation, further corroborating system stability.

**FIGURE S7 |** Dynamic monitoring of flow and pressure during leak testing of the HTHP reactor.

**S8 |** **Detailed Methodology for Calculating CO_2_ Sequestration Capacity**

The relationship between CO_2_ density and pressure was derived from experimental and theoretical data [5−8], as presented in Figure S8. A binomial regression characterized this relationship for the gaseous phase (0–7.39 MPa; Figure S8A), while a trinomial regression was employed for the supercritical phase (7.4–20 MPa; Figure S8B). These fitted correlations were subsequently used to compute the mass of CO_2_ occupying the headspace of the reactor (*V*=50 mL). For example, at a reactor pressure of 5.0 MPa, CO_2_ density is calculated using the fitted function in Figure S8A, with mass derived from the density equation (*ρ*=*m*/*V*); at 10.0 MPa, the fitted function in Figure S8B is used.

**FIGURE S8 |** CO_2_ density as a function of pressure at 45 °C. (A) Evolution of CO_2_ density over the low-to-moderate pressures regime (0.1–7.39 MPa). (B) Pressure-dependent CO_2_ density under elevated pressures (7.4–20 MPa).

**S9 |** **Comparison of Experimental Data with Literature Values**

Figure S9 presents a comparative analysis of CO_2_ solubilities across different pressures, with results from this study (red hollow circles) plotted alongside previously reported data [3] (blue and grey squares). Below the supercritical region (P < 7.39 MPa), the data align closely with established values, validating the consistency and reliability of the experimental methodology.

**FIGURE S9 |** Comparison of CO_2_ solubilities determined in this work with literature data. Literature data were selected based on temperature and pressure conditions matching this study, despite differences in brine concentration. Note: CO_2_ solubility in brine decreases with increasing salinity.

In the supercritical region, however, measurements are significantly higher than literature values. This discrepancy stems from methodological differences: literature studies typically rely on sampling for solubility quantification, where CO_2_ molecules dissolved in the aqueous phase may escape during depressurization, leading to undercounting of total dissolved CO_2_. In contrast, the present approach employs dual mass flowmeters to quantify inlet/outlet CO_2_ mass while continuously monitoring reactor pressure, enabling direct in situ measurement of total CO_2_ content in the sample—explaining the observed variance.

**S10 |** **CO_2_ Sequestration Capacity of Erucamine Solution and Saline Water**

CO_2_ sequestration kinetics of 2.0 wt% erucamine solution, saline water (4500 mg⋅L^−1^ NaCl), and erucamine dispersion of varying concentrations were measured under various CO_2_ pressures. Experimental data in Figure S10 quantify sequestration capacity across these conditions, forming the basis for the comparative analysis discussed in the main text.

**FIGURE S10 |** Kinetics of CO_2_ sequestration by erucamine solution and saline water. (A) CO_2_ uptake kinetics of 2.0 wt% erucamine solution under varying CO_2_ pressures at 45 °C. (B) CO_2_ storage capacity of saline water (4500 mg⋅L^−1^ NaCl) under different CO_2_ pressures at 45 °C. (C) Sequestration kinetics of erucamine dispersion (1.0–5.0 wt%) at 10 MPa CO_2_ and 45 °C.

**S11 |** **Viscosity of Erucamine–DESs Mixed Solutions Before and After CO_2_ Treatment**

Viscosity measurements revealed that CO_2_-induced thickening behavior was retained upon incorporating 5.0 wt% DESs into 3.0 wt% erucamine solution (Figure S11). This result confirms that DESs at this concentration do not compromise the intrinsic CO_2_-responsive properties of the erucamine system.

**FIGURE S11 |** Viscosity evolution of 3.0 wt% erucamine aqueous solution and the corresponding erucamine–DESs mixed solution pre- and post-CO_2_ exposure. The thermal profile reflects the temperature gradient from surface conditions to saline aquifer environments, marking the corresponding change in solution temperature.

**S12 |** **CO_2_ Sequestration Capacity of DESs-Based Solutions**

Raw data for CO_2_ sequestration kinetics—used to quantify the performance of pure DESs and erucamine-DESs mixed solutions under 10 MPa CO_2_ and 45 °C—are presented in Figure S12.

**FIGURE S12 |** CO_2_ uptake kinetics of DESs-containing mixed solutions. (A) Effects of DESs concentration (1.0–10.0 wt%) on CO_2_ sequestration at 45 °C under 10 MPa CO_2_. (B) CO_2_ sequestration behavior of 3.0 wt% erucamine solutions as a function of DESs loading under identical conditions.

**S13 |** **Structural Characterization and Purity Assessment of Erucamine**

Erucamine was synthesized following a previously reported method [9], with structural integrity and purity confirmed *via* comprehensive analytical characterization: ^1^H NMR spectroscopy and high-performance liquid chromatography (HPLC). HPLC separation was performed on a reversed-phase C_18_ column (Waters 515 system, USA) with detection by an Alltech 2000 evaporative light scattering detector.

For ^1^H NMR analysis (Figure S13), data were acquired on a Bruker Avance III HD spectrometer (400 MHz). ^1^H NMR (400 MHz, Chloroform-*d*) δ 6.92 (s, 1H), 5.35 (t, J = 4.8 Hz, 2H), 3.33 (q, J = 5.9 Hz, 2H), 2.38 (t, J = 6.4 Hz, 2H), 2.23 (s, 7H), 2.14 (t, J = 7.6 Hz, 2H), 2.01 (q, J = 6.4 Hz, 4H), 1.62 (dt, J = 22.3, 7.2, 6.7 Hz, 5H), 0.88 (t, J = 6.7 Hz, 3H). These spectra corroborated the expected molecular structure.

**FIGURE S13 |** ^1^H NMR spectrum of erucamine (400 MHz, 25 °C).

HPLC analysis (Waters 515 system, Figure S14) confirmed a purity exceeding 98%—meeting the stringent requirements for subsequent experimental applications.

**FIGURE S14 |** HPLC analysis of erucamine with inset showing peak deconvolution.

**Table S1** Summary of CO_2_ solubilities under different conditions

| **Method** | **Device** | **Method** | **Pressure & temperature** | **Solubility** | **Refs.** |
| --- | --- | --- | --- | --- | --- |
| Simulation | / | Calculation | 10−30 MPa, 40 °C | 10−30% | [10] |
| Simulation | / | Calculation | 20 MPa, 62 °C | 1.6% | [11] |
| Simulation | / | Calculation | 12.4 MPa, 50 °C | 17−25% | [12] |
| Simulation | / | Calculation | 12 MPa, 40 °C | 6% | [13] |
| Simulation | / | Calculation | 6.2 MPa, 37 °C | 3.8% | [14] |
| Experiment | PVT cell | Gasometer | 1.76−20.87 MPa, 59 °C | 0.24−0.96 mmol⋅cm^−3^ | [15] |
| Experiment | HP cell | mass flowmeter | 6.0 MPa, 20−100 °C | 0.007−0.022 mol% | [16] |
| Experiment | HP cell | Gravimetric | 10−20 MPa, 30−60 °C | 1.91−2.39% | [2] |
| Experiment | HP cell | Gravimetric | 15.0 MPa, 50−150 °C | 0.39−1.24% | [17] |
| Experiment | HP cell | Gravimetric | 7.5−15 MPa, 35−70 °C | 4.99−5.80% | [18] |
| Experiment | PVT cell | Gravimetric | 2−16 MPa, 35−55 °C | 1−5% | [3] |
| Experiment | PVT cell | gas analyser | 6.9−22 MPa, 30−140 °C | 0.40−1.20% | [19] |
| Experiment | HP cell | Titration | 7.2~27.2 MPa, 100 °C | 1.1−2.2% | [20] |

**Movie S1.** Sol‒gel phase transition process of a 5.0 wt% erucamine dispersion at 45 °C under 10 MPa CO_2_ visualized using a drop shape analyzer (DSA100 HP1750).

**Movie S2.** Phase transition process of a 5.0 wt% erucamine dispersion at 45 °C from ambient pressure to 10 MPa CO_2_ imaged with a CCD camera on a custom HTHP visualization.

**Movie S3.** Phase transition process of a 5.0 wt% erucamine dispersion at 45 °C upon CO_2_ depressurization from 10 MPa to ambient pressure, recorded by a CCD camera on a custom HTHP visualization platform.

**Movie S4.** Visual documentation of a 5.0 wt% erucamine dispersion before and after carbon sequestration experiment at 10 MPa, captured by a CCD camera.

**References**

1. R. Gupta, R. Mitishita, I. A. Frigaard, G. J. Elfring, “Rheology of Wormlike Micellar Gels Formed by Long-Chained Zwitterionic Surfactants,” *Journal of Rheology* 65 (2021): 1065−80, https://doi.org/10.1122/8.0000285.
2. S. Bando, F. Takemura, M. Nishio, E. Hihara, M. Akai, “Solubility of CO_2_ in Aqueous Solutions of NaCl at (30 to 60) °C and (10 to 20) MPa,” *Journal of Chemical and Engineering Data* 48 (2003): 576−79, https://doi.org/10.1021/je0255832.
3. Y. Liu, M. Hou, G. Yang, B. Han, “Solubility of CO_2_ in Aqueous Solutions of NaCl, KCl, CaCl_2_ and Their Mixed Salts at Different Temperatures and Pressures,” *Journal of Supercritical Fluids* 56 (2011): 125−29, https://doi.org/10.1016/j.supflu.2010.12.003.
4. Z. Zhang, W. Wu, Z. Liu, B. Han, H. Gao, T. Jiang, “A Study of Tri-phasic Behavior of Ionic Liquid–Methanol–CO_2_ Systems at Elevated Pressures,” *Physical Chemistry Chemical Physics 6* (2004): 2352−57, https://doi.org/10.1039/B315417C.
5. I. Johns, S. Rashid, J. T. R. Watson, A. A. Clifford, “Thermal Conductivity of Argon, Nitrogen and Carbon Dioxide at Elevated Temperatures and Pressures,” *Journal of the Chemical Society* 82 (1986): 2235−46, https://doi.org/10.1039/F19868202235.
6. C. Scott, A. I. Johns, J. T. R. Watson, A. A. Clifford, “Thermal Conductivity of Carbon Dioxide in the Temperature Range 300–348 K and Pressures up to 25 MPa,” *Journal of the Chemical Society* 79 (1983): 733−40, https://doi.org/10.1039/F19837900733.
7. R. Span, W. Wagner, “A New Equation of State for Carbon Dioxide Covering the Fluid Region from the Triple‐point Temperature to 1100 K at Pressures up to 800 MPa,” *Journal of Physical and Chemical Reference Data* 25 (1996): 1509−96, https://doi.org/10.1063/1.555991.
8. V. Vesovic, W. A. Wakeham, G. A. Olchowy, J. V. Sengers, J. T. R. Watson, J. Millat, “The Transport Properties of Carbon Dioxide,” *Journal of Physical and Chemical Reference Data* 19 (1990): 763−808, https://doi.org/10.1063/1.555875.
9. Y. Zhang, Z. Chu, C. A. Dreiss, Y. Wang, C. Fei, Y. Feng, “Smart Wormlike Micelles Switched by CO_2_ and Air,” *Soft Matter* 9 (2013): 6217−21, https://doi.org/10.1039/C3SM50913C.
10. L. G. H. van der Meer, “Computer Modelling of Underground CO_2_ Storage,” *Energy Conversion and Management* 37 (1996): 1155−60, https://doi.org/10.1016/0196-8904(95)00313-4.
11. T. Holt, J. I. Jensen, E. Lindeberg, “Underground Storage of CO_2_ in Aquifers and Oil Reservoirs,” *Energy Conversion and Management* 36 (1995): 535−38, https://doi.org/10.1016/0196-8904(95)00061-H.
12. D. H. S. Law, S. Bachu, “Hydrogeological and Numerical Analysis of CO_2_ Disposal in Deep Aquifers in the Alberta Sedimentary Basin,” *Energy Conversion and Management* 37 (1996): 1167−74, https://doi.org/https://doi.org/10.1016/0196-8904(95)00315-0.
13. K. Pruess, T. Xu, J. Apps, J. Garcia, “Numerical Modeling of Aquifer Disposal of CO_2_,” *SPE Journal* 8 (2003): 49−60, https://doi.org/10.2118/83695-PA.
14. S. Ghanbari, Y. Al-Zaabi, G. E. Pickup, E. Mackay, F. Gozalpour, A. C. Todd, “Simulation of CO_2_ Storage in Saline Aquifers,” *Chemical Engineering Research & Design* 84 (2006): 764−75, https://doi.org/10.1205/cherd06007.
15. Z. Li, M. Dong, S. Li, L. Dai, “Densities and Solubilities for Binary Systems of Carbon Dioxide + Water and Carbon Dioxide + Brine at 59 °C and Pressures to 29 MPa,” *Journal of Chemical and Engineering Data* 49 (2004): 1026−31, https://doi.org/10.1021/je049945c.
16. F. Lucile, P. Cézac, F. Contamine, J. Serin, D. Houssin, P. Arpentinier, “Solubility of Carbon Dioxide in Water and Aqueous Solution Containing Sodium Hydroxide at Temperatures from (293.15 to 393.15) K and Pressure up to 5 MPa: Experimental Measurements,” *Journal of Chemical and Engineering Data* 57 (2012): 784−89, https://doi.org/10.1021/je200991x.
17. H. Zhao, M. V. Fedkin, R. M. Dilmore, S. N. Lvov, “Carbon Dioxide Solubility in Aqueous Solutions of Sodium Chloride at Geological Conditions: Experimental Results at 323.15, 373.15, and 423.15 K and 150 Bar and Modeling Up to 573.15 K and 2000 Bar,” *Geochemica et Cosmochimica Acta* 149 (2015): 165−89, https://doi.org/10.1016/j.gca.2014.11.004.
18. G. Ferrentino, D. Barletta, F. Donsì, G. Ferrari, M. Poletto, “Experimental Measurements and Thermodynamic Modeling of CO_2_ Solubility at High Pressure in Model Apple Juices,” *Industrial & Engineering Chemistry Research* 49 (2010): 2992−3000, https://doi.org/10.1021/ie9009974.
19. K. Gilbert, P. C. Bennett, W. Wolfe, T. Zhang, K. D. Romanak, “CO_2_ Solubility in Aqueous Solutions Containing Na^+^, Ca^2+^, Cl^−^, SO_4_^2−^ and HCO_3_^−^: The Effects of Electrostricted Water and Ion Hydration Thermodynamics,” *Applied Geochemistry* 67 (2016): 59−67, https://doi.org/10.1016/j.apgeochem.2016.02.002.
20. J. Qin, R. J. Rosenbauer, Z. Duan, “Experimental Measurements of Vapor–Liquid Equilibria of the H_2_O + CO_2_ + CH_4_ Ternary System,” *Journal of Chemical and Engineering Data* 53 (2008): 1246−49, https://doi.org/10.1021/je700473e.
